# Structural, Electrical and Optical Properties of Pyrrolo[1,2-*i*][1,7] Phenanthroline-Based Organic Semiconductors

**DOI:** 10.3390/ma15051684

**Published:** 2022-02-23

**Authors:** Corneliu Doroftei, Aurelian Carlescu, Liviu Leontie, Ramona Danac, Cristina Maria Al-Matarneh

**Affiliations:** 1 Research Center in Environmental Sciences for the North-Eastern Romanian Region (CERNESIM), Science Research Department, Institute of Interdisciplinary Research, Alexandru Ioan Cuza University of Iasi, Bulevardul Carol I, Nr. 11, 700506 Iasi, Romania; corneliu.doroftei@uaic.ro (C.D.); lleontie@uaic.ro (L.L.); 2 Faculty of Physics, Alexandru Ioan Cuza University of Iasi, Bulevardul Carol I, Nr. 11, 700506 Iasi, Romania; 3 Faculty of Chemistry, Alexandru Ioan Cuza University of Iasi, Bulevardul Carol I, Nr. 11, 700506 Iasi, Romania; rdanac@uaic.ro (R.D.); almatarneh.cristina@yahoo.ro (C.M.A.-M.); 4 Institute of Macromolecular Chemistry “Petru Poni”, Aleea Ghica Voda, No. 41A, 700487 Iasi, Romania

**Keywords:** organic semiconductors, pyrrolo-phenathrolines, thin films, electrical properties, optical properties

## Abstract

This work reports a study on structural, electrical and optical properties of some recently synthesized pyrrolo[1,2-*i*][1,7] phenanthrolines derivatives in thin films. The thin films were deposited onto glass substrates by spin coating technique, using chloroform as solvent. The obtained films exhibited a polycrystalline structure with an *n*–type semiconductor behavior after heat treatment in the temperature range 293–543 K, specific to each sample. The thermal activation energy lies between 0.68 and 0.78 eV, while the direct optical band gap values were found in the range 4.17–4.24 eV. The electrical and optical properties of the investigated organic semiconductor films were discussed in relation to microstructural properties, determined by the molecular structure. The investigated organic compounds are promising for applications in organic optoelectronics and nanoelectronics.

## 1. Introduction

In recent years, a special emphasis has been directed toward the development of new organic semiconductor materials for a broad spectrum of applications. Electronics based on organic semiconductors offer new intriguing opportunities, some of which cannot be reached by inorganic electronics. Organic electronics enables the building of inexpensive, high-performance and large-area devices using low-temperature techniques compatible with flexible devices, thus enabling the exploitation in a broad range of new applications [1,2,3,4]. Due to their small molecular weight (under 1000 g/mol) [5], mechanical flexibility, reduced production costs, low processing temperature (typically below 100 °C) [6], solubility (in polar or nonpolar solvents, depending on molecular structure), as well as abundance, they exhibit a high level of manufacturability (spin coating, reel-to-reel fabrication, ink-jet printing, evaporation) and versatility, are compatible with flexible substrates and easily integrate with diverse physicochemical and biological functions [7,8,9]. These materials show potential for multiple applications: organic light-emitting diodes for display applications and lighting devices, electronic paper, supercapacitors, thin-film fuel cells, organic photovoltaics, lasers, sensors and biosensors, as well as in nanoelectronics, optoelectronics, transparent electronics and spintronics [10,11,12,13].

Thus, low-molecular-weight organic semiconductors, especially in the form of thin films with controllable structural and physical characteristics, are often preferable to single-crystalline ones in many (opto)electronic applications [14]. Furthermore, the mobility of charge carriers in active low-molecular-weight organics, which substantially affects the performance of organic (opto)electronic devices, is much higher (roughly one order of magnitude) than that of polymer semiconductors, because of a more effective control of electron transfer properties, facilitated by chemical tunability, mainly in the case of polycrystalline thin films [15,16].

Being suitable for molecular tailoring, a synthesis of over twenty million organic compounds has been reported so far [17]. A series of notable physical characteristics related to electroluminescence, high mobility of charge carriers (higher than 10 cm^2^ V^−1^ s^−1^) [18], energy band gap (typically of 2.5–4 eV) in the IR–Vis domain, etc., combined with their chemical adjustability, give these materials the ability to cover an extended range of technological applications. A number of devices have already been developed and commercialized: flexible displays, smart cards, radio-frequency identification tags, solid state lighting, etc. [19,20].

Continuing our research focused on the synthesis and characterization of new fused N-heterocyclic functional compounds [21,22,23,24,25,26,27,28], we present in this paper the investigation of microstructural, electrical and optical properties of a series of pyrrolo[1,2-*i*][1,7] phenanthroline derivatives LL(1–3), in the form of thin films.

## 2. Materials and Methods

### 2.1. Synthesis of Compounds

**Scheme 1:**
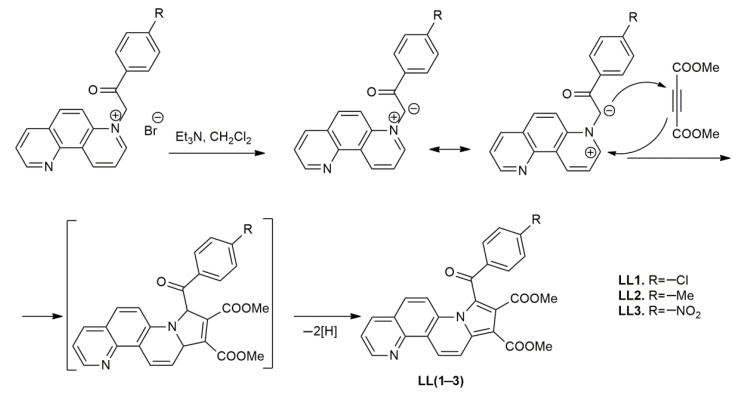
Synthesis pathway of the investigated compounds (**LL1**, **LL2** and **LL3**).

Indolizine derivatives with 1,7-phenanthroline skeleton LL(1–3) have been recently synthesized [29] using, as a key step, a Huisgen [3+2] dipolar cycloaddition of the N-ylides generated in situ, starting from the monoquaternary 1,7-phenanthrolin-7-ium salts [30] in basic medium (triethylamine). The ylidic intermediates play 1,3-dipole roles when reacting with dipolarophyles with activated double or triple bond. In reaction with dimethylacetylene dicarboxylate (DMAD) as symmetric substituted dipolarophile, unisolable cycloadducts with dihydropyrrolo[1,2-*i*][1,7] phenanthroline structure were first generated, but under reaction conditions, they undergo a spontaneous oxidation to yield derivatives LL(1–3) (Scheme 1).

The structure of the compounds (together with the type of substituents) is presented along with their melting points, in Scheme 1 and Table 1. The products were completely characterized using spectral (IR (Infrared), 1H- and 13C-NMR (Nuclear Magnetic Resonance), Mass Spectrometry) and analytical techniques [29,30,31]. Organic compounds under study were synthesized in the form of polycrystalline powders and were found to exhibit good chemical stability in ambient atmospheric environment.

### 2.2. Thin Films Preparation and Measurement Setup

In order to study their structural, morphological, electrical and optical characteristics, compounds LL(1–3) were deposited in thin films (surface-type cells) using the spin coating technique [32,33,34,35,36] on top of glass substrates, at room temperature.

The corresponding pyrrolo[1,2-*i*][1,7] phenanthroline powder was dissolved in chloroform, yielding the solution from which the thin films were grown. In order to obtain films with a uniform thickness, we used solutions with concentrations ranging between 2 and 10 mg/mL, a substrate spinning speed of 1500 rpm, 5–8 coating and drying cycles at a temperature equal to 50–60 °C and a heat treatment at about 10 °C below the melting point (Table 1).

The thickness of the obtained films (10–13 µm) was determined using a MII-4 interferometric microscope (LOMO, St. Petersburg, Russia).

Microstructural characterization of the organic thin films was performed using X-ray diffraction (XRD) technique (a Shimadzu LabX XRD 6000 diffractometer, with a CuKα radiation source (wavelength *λ* = 1.54182 Ǻ)). The morphology of thin-film sample surface was investigated using atomic force microscopy (AFM) (a NT-MDT Solver Pro m type microscope).

To determine the d.c. electrical properties, glass substrates with parallel conductive silver electrodes previously deposited were used. The contact between the electrode surface and the deposited organic film was achieved without forming a junction. Temperature dependence of the electrical conductivity of thin wires was determined by the two-probe method, using a device equipped with a temperature-adjustable electrical heater on which the sample was placed, with two electrode contacts and a KEITHLEY Model 6517B electrometer.

To determine the optical properties of organic films deposited on glass, a STEAK-ETA-OPTIK spectrometer operating in the UV–Vis–NIR field was used.

## 3. Results and Discussions

**Figure 1:**
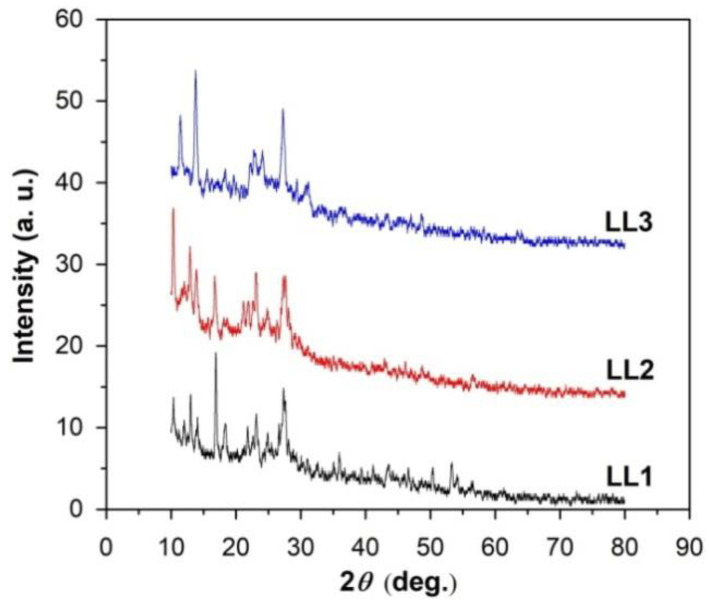
XRD patterns for the organic films under study.

From the X-ray diffraction analyses, it can be inferred that the studied organic films exhibit a polycrystalline structure that differs depending on the sample nature (the position and nature of the R substituents within the organic molecules). Figure 1 shows the X-ray diffractograms for the 2*θ* domain between 10 and 80 deg.

Bragg angles (2*θ*) were identified from the X-ray diffractograms for the most intense peaks of the samples, which subsequently led to the calculation of the interplanar distances (*d_hkl_*) and average crystallite sizes (*D*).

Thus, the interplanar distances (*d_hkl_*) were determined using the Bragg equation [37,38]:

(1) $$2d_{hkl}\sin\theta =n\lambda$$

where *θ* is the Bragg diffraction angle, *λ* is the incident X-ray wavelength (*λ* = 1.54182 Ǻ), *n* denotes the order of reflection (*n* = 1), and *h*, *k*, *l* represent the Miller indices.

The average crystallite sizes (*D*) were determined using the Debye–Scherrer equation [39,40,41]:

(2) $$D=\frac{0.9\lambda }{\beta \cos\theta }$$

where *λ* is the radiation wavelength as specified above, *β* is the full-width-at-half maximum of the peak, and *θ* is the Bragg diffraction angle.

The values for Bragg angles, interplanar distances and crystallite sizes for the studied organic films are given in Table 2.

The XRD analyses (Figure 1 and Table 2) also revealed that certain values of the interplanar distances are common for the studied compounds. Additionally, other values of the interplanar distances caused by the distinct nature of the substituents (R) can be emphasized, which is also manifested in the values of the crystallite sizes. The average crystallite size was found to decrease from 42 nm for compound LL1 (R = −Cl) to 26 nm for compound LL2 (R = −Me) and to 22 nm for compound LL3 (R = −NO2).

**Figure 2:**
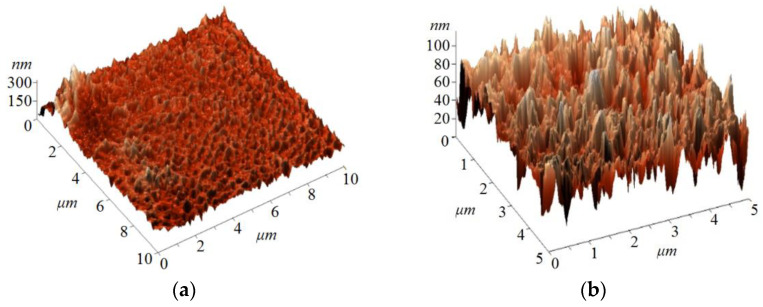
3D AFM images for the organic film **LL1**, scale 10 × 10 mm^2^ (**a**) and scale 5 × 5 mm^2^ (**b**).

Films with thickness in the range of 10–13 μm were obtained (Table 3), showing a surface with good uniformity, without precipitates or holes. The films comprised granules of different sizes and shapes, with their base on the substrate surface. Surface roughness varied insignificantly with film composition. The average roughness (Ra) of the obtained films was around 17 nm, the root mean square roughness (Rrms) around 24 nm, and the size of the polycrystals was in the range of 10–75 nm. Figure 2 shows the 3D AFM images for the organic thin film LL1, performed at different scales.

Film thickness, deposition conditions and molecular structures are the main factors affecting the structure particularities of the samples. Microstructural properties play an important role in the electronic transport mechanism in semiconducting organic compounds [4,17]. As-synthesized organic films are characterized by a polycrystalline structure, which plays a key role in electronic conduction mechanisms in actual organic thin films. Besides, the possibility of lower values for electrical conductivity and higher values for charge carrier mobilities due to the scattering mechanisms on the sample surface and at the crystallite boundaries, is known to be very common in thin films samples with a polycrystalline structure [42,43].

The electrical conductivity (*σ*) of the films was found to increase exponentially with temperature in the studied temperature range (Table 3), according to the known law [44,45,46,47]:

(3) $$\sigma =\sigma_{0}\exp\left(-\frac{E_{a}}{k_{B}T}\right)$$

where *E_a_* denotes the activation energy for d.c. conductivity, *σ_0_* is a characteristic parameter, which depends on the nature of the sample, and *k_B_* represents the Boltzmann’s constant.

In the measured temperature range, based on the band model representation, two distinct conduction regimes can be detected: extrinsic and intrinsic. These two temperature domains are delimited by the *T_c_*, which is a characteristic temperature for each sample (Table 3). According to the mentioned model, in the intrinsic conduction domain (higher temperature range), thermal activation energy *E_a_* (*E_a_* = *E_g_*/2) is half of band gap energy value of the material [48], representing the energy difference between lower occupied energy level with electrons from the conduction band (CB) and higher energy level occupied by holes from valence band (VB).

Additionally, in temperature range of the extrinsic conduction regime (*T* < *T_c_*), it signifies the energy of donor levels relative to the CB bottom, or that of the acceptor levels relative to the VB top, respectively [44,45,46].

**Figure 3:**
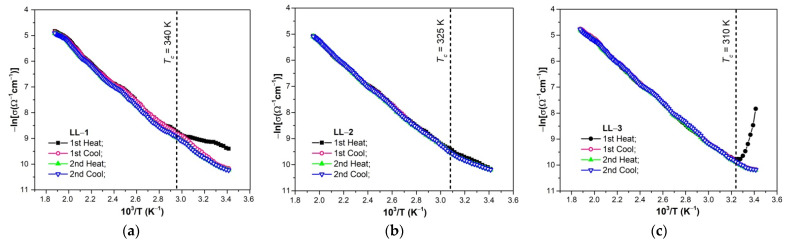
Temperature dependence of electric conductivity of the studied organic films ((**a**) **LL1**; (**b**) **LL2**; (**c**) **LL3**) for two heating/cooling complete cycles.

In Figure 3, the conductivity–temperature characteristics (ln*σ* = f(10^3^/*T*)) for the organic thin films in the measured temperature range, Δ*T*, for two heating/cooling complete cycles are shown. 

For the first heating (Figure 3), in the temperature range of 20–60 °C, a deviation from the law (3) of the electrical conductivity of the samples can be found, and this may be due to the influence of the environmental humidity or other (chemical) factors [49,50,51,52]. Practically, the characteristics do not change considerably due to repeated heating and cooling. It can be said that the studied films present stable structure and reversible electrical conductivity.

The ln*σ* vs. 10^3^/*T* characteristics (Figure 3a–c) show two regions with different slopes corresponding to the mentioned different temperature ranges. In lower temperature range (*T* < *T_c_*), we found a smaller slope, compared to that for the higher temperature region (*T* > *T_c_*). This reveals that there are two thermal activation energies, which implies two electrical conduction mechanisms operating in the respective temperature ranges. These characteristics are typical for the wide band gap semiconductors.

The thermal activation energy (*E_a_*) was determined using the slope of ln*σ* = f(10^3^/*T*) characteristics corresponding to the intrinsic conduction region (in the higher temperature domain). The values of thermal activation energies thus determined are in the range of 0.68–0.78 eV (Table 3) and are specific for semiconductors.

From the analysis of the characteristics presented in Figure 3, it can be deduced that the band model representation can be properly used to investigate the electron transfer mechanisms in actual organic semiconductor thin films, in higher temperature interval (*T* > *T_c_*), which corresponds to intrinsic conduction regime.

The semiconductor behavior of the organic compounds under study is determined by their microstructure and molecular configuration. We performed the Hall measurements and determined that the organic compounds studied exhibit an *n*–type semiconductor behavior. The electron systems within the samples are very sensitive to the modifications brought by the transport processes that determine excitations and addition/removal of charge carriers. These electronic configurations typically contain π electrons (because of aromatic rings) and allow the formation of highly delocalized wave functions along the molecular backbone, especially on the pyrrolo[1,2-*i*][1,7] phenanthroline fused skeleton. If the number of *π*-electrons increases, this would lead to a decrease in thermal activation energy, as well as an increase in electrical conductivity [53,54]. The presence of extended conjugation systems in the studied organic thin films stimulates the electron transfer and electrical conduction inside molecules. The value of thermal activation energy can be manipulated through the position and nature of the substituents (*R*) in the molecules that determine the degree of conjugation (Scheme 1). Compared to other organic semiconductors with similar structure, actual compounds exhibit lower thermal activation energy, but higher electrical conductivity [44,45]. Moreover, the studied compounds show a good stability in the considered temperature range (Table 3).

**Figure 4:**
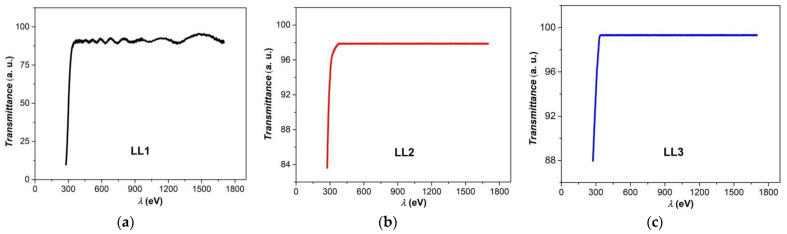
Transmittance spectra of investigated organic films ((**a**) **LL1**; (**b**) **LL2**; (**c**) **LL3**).

The actual organic semiconductors obtained in the form of thin films were also investigated, regarding the optical properties. With the help of a STEAG ETA-Optik spectrometer, two series of data were measured: transmittance (*T*) and reflectance (*R*), respectively, as functions of wavelength (*λ*), in a spectral range between 300 and 1700 nm (Vis–NIR). For this wavelength range, the films display transmittance values of over 90% (Figure 4a–c) and reflectance values of less than 10%.

Using Equation (4) [55]

(4) $$\alpha =\frac{1}{d}\ln[\left(1-R^{2}\right)/T$$

where *d* represents thin-film thickness, *T* is transmittance, and *R* is reflectance, the absorption coefficient (*α*) was determined.

**Figure 5:**
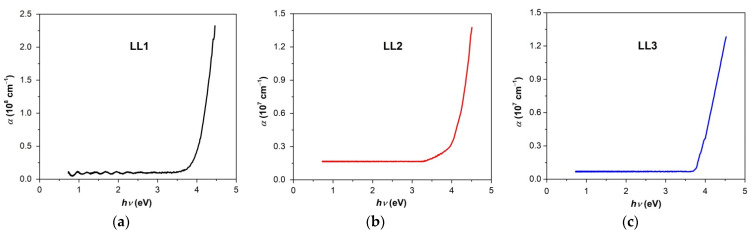
Spectral dependence of the absorption coefficient for organic films under study ((**a**) **LL1**; (**b**) **LL2**; (**c**) **LL3**).

Figure 5a–c presents the spectra of the absorption coefficient (*α*) (its dependence on the photon energy (*hν*)) of the organic films under study. The energy of incident photon was determined with the help of the relation (5):

*E* = *hν* = 1.2398/*λ* (5)

where *λ* is incident wavelength in nm, and *E* is expressed in eV.

In the vicinity of fundamental absorption edge, the absorption coefficient (*α*) varies with energy of the incident photon, according to [56,57]

*αhν* = *A*(*hν* − *E_go_*)*^n^* (6)

where *n* is 1/2 or 2 for optical direct or indirect allowed transitions, respectively, *E_go_* is the optical band gap, direct (*E_go_^d^*) or indirect (*E_go_^i^*), and *A* is a characteristic parameter, not dependent on the photon energy.

The refractive index was determined using Equation (7) [58]:

(7) $$n_{r}=\frac{(1+R)+\sqrt{4\cdot R-{\left(1-R\right)}^{2}\cdot k^{2}}}{1-R}$$

where *R* denotes reflectance, *k* is the extinction coefficient (*k* = *αλ*/4*π*), α is the absorption coefficient, and *λ* is the wavelength.

**Figure 6:**
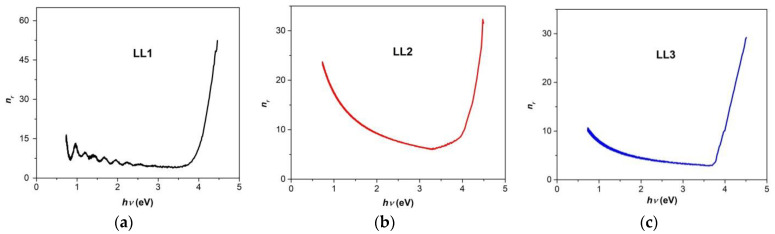
Refractive index as a function of photon energy (*hν*) for investigated organic films ((**a**) **LL1**; (**b**) **LL2**; (**c**) **LL3**).

Figure 6a–c shows the spectra of the refractive index (*n_r_*) for the studied organic films.

Equation (6) can be written as [55,59]

(8) $$\frac{d\left[\ln\left(\alpha h\nu \right)\right]}{d\left[h\nu \right]}=\frac{n}{h\nu -E_{go}}$$

**Figure 7:**
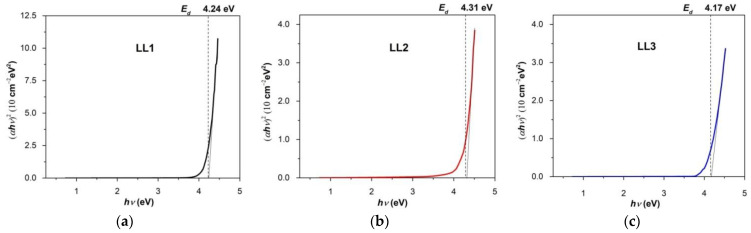
Optical absorption spectra of present organic films, emphasizing direct optical band gaps of organic films ((**a**) **LL1**; (**b**) **LL2**; (**c**) **LL3**).

The type of electron transition can be determined according to the value of *n*, found by plotting the dependence *d*[ln(*αhν*)]/*d*(*hν*) versus *hν* [59,60,61,62]. Thus, to determine the optical band gap (*E_go_*), we plotted experimental (*αhν*)^2^ = *f*(*hν*) dependence (Figure 7a–c). The optical band gap was determined by extrapolation of the linear part of the plot to zero absorption. This indicates that direct allowed transitions play a key role in determining the nature of the fundamental absorption edge of actual films. Determined values for the direct band gaps (*E_go_^d^*) were of 4.24 eV, 4,31 eV and 4.17 eV for the studied organic films, LL1, LL2 and LL3, respectively.

The magnitude of *E_a_* (activation energy, Table 3) obtained from conductivity measurements is small compared to the half of *E_go_* (optical band gap energy). This fact is due to quite a different nature of the carrier excitation in the involved processes (electric conduction and optical absorption). The values of *E_go_^d^* correspond to direct (band-to-band) transitions, whereas those of *E_a_* are determined by the prevailing electron transfer mechanism in present organic films.

The obtained values for thermal activation energy and optical band gap of present organic films are comparable with those obtained in case of several recently reported series of fused heterocyclic compounds (polycrystalline thin films) that contain 1,7-fenanthroline core [30], indolizine skeleton [63] or pyridinium disubstituted ylides [14].

## 4. Conclusions

The microstructural, electrical and optical properties of some recently synthesized organic compounds, with pyrrolo[1,2-*i*][1,7] phenanthroline structure in the form of thin films, were studied. The films display a polycrystalline structure that differs depending on the sample composition (the nature of the R substituents within the organic molecules). The compounds exhibit a *n*–type semiconductor behavior. The values of thermal activation energies are specific to semiconductors and are in the range of 0.68–0.78 eV. The band model representation can be successfully used to investigate the electron transfer mechanisms in actual organic semiconductor thin films in the higher temperature range (*T* > *T_c_*, the intrinsic domain). In the spectral range 300–1700 nm, the films have a transmittance of over 90% and reflectance values of less than 10%. The optical band gap (direct) has values in the range of 4.17–4.24 eV. The microstructural properties, determined by the molecular structure that differs only by the nature of para-substituent (R) of the benzene ring, were correlated with the electrical and optical properties of compounds. The investigated organic compounds are promising for applications in nanoelectronics, and especially in organic optoelectronics.

## Figures and Tables

**Table 1:** Molecular structure and some characteristics of examined compounds.

Compound	Molecular Formula	Molecular Weight, *M* (g/mol)	Color	Melting Point (°C)
**LL1**	C_26_H_17_ClN_2_O_5_	472.88	orange	270–271
**LL2**	C_27_H_20_N_2_O_5_	452.46	yellow	245–247
**LL3**	C_26_H_17_N_3_O_7_	483.43	orange	284–286

**Table 2:** Structural parameters obtained from X-ray analysis of the studied samples (**LL1**, **LL2** and **LL3**).

LL1				LL2				LL3			
*I*/*I*_0_ (%)	2*θ* (deg.)	*d_hkl_* (nm)	*D* (nm)	*I*/*I*_0_ (%)	2*θ* (deg.)	*d_hkl_* (nm)	*D* (nm)	*I*/*I*_0_ (%)	2*θ* (deg.)	*d_hkl_* (nm)	*D* (nm)
33.23	10.34	0.803	59.5	100	10.37	0.801	27.77	51.4	11.41	0.728	23.16
7.15	10.83	0.767	75.76	21.57	12	0.692	41.71	100	13.76	0.604	21.42
9.61	11.24	0.739	41.68	62.04	12.9	0.644	27.83	21.46	15.53	0.535	18.6
24.07	12.01	0.692	36.27	51.9	13.9	0.598	23.88	9.5	16.29	0.511	25.39
49.69	13.01	0.639	49.12	18.97	15.7	0.53	41.87	5.67	16.67	0.499	29.94
30.07	14.05	0.592	39.81	67.67	16.74	0.497	23.96	18.59	18.36	0.453	23.34
100	16.89	0.493	49.34	27.2	21.15	0.394	28.13	12.44	22.22	0.375	27.27
36	18.34	0.454	20.49	28.42	21.88	0.381	20.12	20.71	22.69	0.368	18.39
27.38	21.81	0.382	42.24	63.34	23.1	0.361	22.28	24.4	24.06	0.347	14.88
15.15	22.63	0.369	42.3	31.02	24.78	0.337	13.7	69.99	27.23	0.307	16.41
37	23.16	0.36	28.23	22.18	26.32	0.318	25.82	20.77	29.44	0.284	32.99
21.84	24.89	0.335	42.48	69.58	27.28	0.307	9.59	22.82	31.16	0.269	10.37
23.23	26.64	0.314	60.9	17.67	46.16	0.184	33.4	12.3	43.3	0.196	14.39
58.23	27.34	0.306	21.34	20.27	48.78	0.175	35.04	12.3	43.32	0.196	14.87
34.76	35.92	0.234	54.51	14.81	56.53	0.152	16.82	15.37	47	0.181	36.19
15.31	39.31	0.215	31.46					17.42	48.66	0.175	21.68
18.38	41.16	0.205	68.17								
17.69	43.54	0.195	17.51								
19.61	46.64	0.182	39.28								
21.38	50.38	0.17	26.96								
26.15	53.31	0.161	27.3								

*I*/*I*_0_—relative integrated intensity; *θ*—Bragg diffraction angle; *d_hkl_*—interplanar distance for adjacent lattice planes with *h*, *k*, *l* Miller indices; *D*—average crystallite size.

**Table 3:** Results of electronic transport measurements.

Compound	*d* (μm)	*σ_c_* (Ω^−1^⋅cm^−1^)	Δ*T* (K)	*σ_T_* (Ω^−1^⋅cm^−1^)	*T_c_* (K)	*E_a_* (eV)	*E_g_* (eV)
**LL1**	10	1.92 × 10^−4^	293–533	7.36 × 10^−3^	340	0.74	1.48
**LL2**	13	3.85 × 10^−3^	293–513	6.17 × 10^−3^	325	0.77	1.54
**LL3**	10	3.95 × 10^−4^	293–543	9.40 × 10^−3^	310	0.76	1.52

## Data Availability

The data presented in this study are available on request from the corresponding author.

## References

[1] He Z., Zhang Z., Bi S. (2020). Nanoparticles for organic electronics applications. Mater. Res. Express.

[2] Lee E.K., Lee M.Y., Park C.H., Lee H.R., Oh J.H. (2017). Toward Environmentally Robust Organic Electronics: Approaches and Applications. Adv. Mater..

[3] Kim K.-H., Park M.-J., Kim J.-H. (2020). Crack-Assisted Charge Injection into Solvent-Free Liquid Organic Semiconductors via Local Electric Field Enhancement. Materials.

[4] Yang J., Yan D., Jones T.S. (2015). Molecular Template Growth and Its Applications in Organic Electronics and Optoelectronics. Chem. Rev..

[5] Kampen T.U. (2010). Low Molecular Weight Organic Semiconductors.

[6] Sirringhaus H. Low-temperature, solution-processed organic transistors for flexible electronics. Proceedings of the IEEE Technology Time Machine Symposium on Technologies Beyond.

[7] Liang Z., Yan L., Si J., Gong P., Li X., Liu D., Li J., Hou X. (2021). Rational Design and Characterization of Symmetry-Breaking Organic Semiconductors in Polymer Solar Cells: A Theory Insight of the Asymmetric Advantage. Materials.

[8] Perinot A., Giorgio M., Mattoli V., Natali D., Cairon M. (2021). Organic Electronics Picks Up the Pace: Mask-Less, SolutionProcessed Organic Transistors Operating at 160 MHz. Adv. Sci..

[9] Zhou Y., Fuentes-Hernandez C., Shim J., Meyer J., Giordano A.J., Li H., Winget P., Papadopoulos T., Cheun H., Kim J. (2012). A Universal Method to Produce Low–Work Function Electrodes for Organic Electronics. Science.

[10] Sun S.-S., Sariciftci N.S. (2005). Organic Photovoltaics: Mechanisms, Materials, and Devices.

[11] Tavasli A., Gurunlu B., Gunturkun D., Isci R., Faraji S. (2022). A Review on Solution-Processed Organic Phototransistors and Their Recent Developments. Electronics.

[12] Yousaf J., Almajali E., El Najjar M., Amir A., Altaf A., Elahi M., Alja’afreh S.S., Rmili H. (2022). *Flexible,* Fully Printable, and Inexpensive Paper-Based Chipless Arabic Alphabet-Based RFID Tags. Sensors.

[13] Amiri M., Shul G., Donzel N., Bélanger D. (2021). Aqueous electrochemical energy storage system based on phenanthroline- and anthraquinone-modified carbon electrodes. Electrochim. Acta.

[14] Danac R., Leontie L., Girtan M., Prelipceanu M., Graur A., Carlescu A., Rusu G.I. (2014). On the d.c. electric conductivity and conduction mechanism of some stable disubstituted 4-(4-pyridyl)pyridinium ylides in thin films. Thin Solid Films.

[15] Karl N. (2003). Charge carrier transport in organic semiconductors. Synth. Met..

[16] Kazheva O.N., Kushch N.D., Aleksandrov G.G., Dyachenko O.A. (2002). Crystal structure and molecular packing in a new organic semiconductor penta[bis(ethylenedithio)tetrathiafulvalene]-hexathiocyanatomononitratoyttrium(III)-ethanol. Mater. Chem. Phys..

[17] Myers R. (2003). The Basics of Chemistry.

[18] Hu P., He P., Jiang H. (2021). Greater than 10 cm^2^V^−1^s^−1^: A breakthrough of organicsemiconductors for field-effect transistors. InfoMat.

[19] Egginger M., Bauer S., Schwodiauer R., Neugebauer H., Sariciftci N.S. (2009). Current versus gate voltage hysteresis in organic field effect transistors. Monatsh. Chem..

[20] Hadis M. (2003). Advanced Semiconductor and Organic Nano-Techniques.

[21] Lu G. (2006). Organic Semiconductor.

[22] Zhang Y., Guo L., Zhu X., Sun X. (2020). The Application of Organic Semiconductor Materials in Spintronics. Front. Chem..

[23] Leontie L., Danac R., Druta I., Carlescu A. (2010). Electron transport properties of some newly synthesized nonsymmetrical bisindolizines in thin films. Synth. Metals.

[24] Prelipceanu M., Prelipceanu O.S., Leontie L., Danac R. (2007). Photoelectron spectroscopy investigations of pyrrolo[1,2-a][1,10] phenanthroline derivatives. Phys. Lett. A.

[25] Popovici L., Amarandi R.M., Mangalagiu I.I., Mangalagiu V., Danac R. (2019). Synthesis, molecular modelling and anticancer evaluation of new pyrrolo[1,2-b] pyridazine and pyrrolo[2,1-a] phthalazine derivatives. J. Enz. Inhib. Med. Chem..

[26] Airinei A., Tigoianu R., Danac R., Al Matarneh C.M., Isac D.L. (2018). Steady state and time resolved fluorescence studies of new indolizine derivatives with phenanthroline skeleton. J. Lumin..

[27] Marangoci N.-L., Popovici L., Ursu E.-L., Danac R., Clima L., Cojocaru C., Coroaba A., Neamtu A., Mangalagiu I.I., Pinteala M. (2016). Pyridyl-indolizine derivatives as DNA binders and pH-sensible fluorescent dyes. Tetrahedron.

[28] Al Matarneh C.M., Mangalagiu I.I., Shova S., Danac R. (2016). Synthesis, structure, antimycobacterial and anticancer evaluation of new pyrrolo-phenanthroline derivatives. J. Enz. Inhib. Med. Chem..

[29] Danac R., Al Matarneh C.M., Shova S., Daniloaia T., Balan M., Mangalagiu I.I. (2015). New indolizines with phenanthroline skeleton: Synthesis, structure, antimycobacterial and anticancer evaluation. Bioorg. Med. Chem..

[30] Leontie L., Danac R., Apetroaei N., Rusu G.I. (2011). Study of electronic transport properties of some new N-(p-R-phenacyl)-1,7-phenanthrolinium bromides in thin films. Mat. Chem. Phys..

[31] Kuki Á., Nagy M., Nagy L., Zsuga M., Kéki S. (2010). Ligand Size Distribution of Phenanthroline—Functionalized Polyethylene Glycol-Iron(II) Complexes Determined by Electrospray Ionization Mass Spectrometry and Computer Simulation. J. Am. Soc. Mass Spectrom..

[32] Zhang S., Audebert P., Wei Y., Al Choueiry A., Lanty G., Bréhier A., Galmiche L., Clavier G., Boissière C., Lauret J.S. (2010). Preparations and Characterizations of Luminescent Two Dimensional Organic-inorganic Perovskite Semiconductors. Materials.

[33] Zhang Y., Wu J., Song J., Chen Z., He J., Wang X., Liu H., Chen S., Qu J., Wong W.-Y. (2019). Achieving High-Performance Solution-Processed Deep-Red/Near-Infrared Organic Light-Emitting Diodes with a Phenanthroline-Based and Wedge-Shaped Fluorophore. Adv. Electron. Mater..

[34] Corriu R., Anh N.T. (2009). Molecular Chemistry of Sol-Gel Derived Nanomaterials.

[35] Hu Z., Miao J., Li T., Liu M., Murtaza I., Meng H. (2018). Reduced interface losses in inverted perovskite solar cells by using a simple dual-functional phenanthroline derivative. Nano Energy.

[36] Sakka S. (2004). Handbook of Sol-Gel Science and Technology: Processing, Characterization and Applications.

[37] Chang Y., Wang H., Zhu Q., Luo P., Dong S. (2013). Theoretical calculation and analysis of ZrO_2_ spherical nanometer powders. J. Adv. Ceram..

[38] Saleem H., Haneef M., Abbasi H.Y. (2018). Synthesis route of reduced graphene oxide via thermal reduction of chemically exfoliated graphene oxide. Mater. Chem. Phys..

[39] Klung H., Alexander L. (1962). X-ray Diffraction Procedures.

[40] Leontie L., Doroftei C. (2017). Nanostructured spinel ferrites for catalytic combustion of gasoline vapors. Catal. Lett..

[41] Doroftei C., Leontie L. (2021). Nanocrystalline SrMnO_3_ perovskite prepared by sol–gel self-combustion method for sensor applications. J. Sol-Gel Sci. Technol..

[42] Brenner T.M., Egger D.A., Kronik L., Hodes G., Cahen D. (2016). Hybrid organic—Inorganic perovskites: Low-cost semiconductors with intriguing charge-transport properties. Nat. Rev. Mater..

[43] Schweicher G., Olivier Y., Lemaur V., Geerts Y.H. (2013). What Currently Limits Charge Carrier Mobility in Crystals of Molecular Semiconductors?. Isr. J. Chem..

[44] Satyavani T.V.S.L., Kiran B.R., Kumar V.R., Kumar A.S., Naidu S.V. (2016). Effect of particle size on dc conductivity, activation energy and diffusion coefficient of lithium iron phosphate in Li-ion cells. Int. J. Eng. Sci. Technol..

[45] Yoshino T., Shimojuku A., Shan S., Guo X., Yamazaki D., Ito E., Higo Y., Funakoshi K. (2012). Effect of temperature, pressure and iron content on the electrical conductivity of olivine and its high-pressure polymorphs. J. Geophys. Res..

[46] Baraker B.M., Lobo B. (2017). Analysis of Electrical Measurements on Cadmium Chloride Doped PVA-PVP Blend. Mapana J. Sci..

[47] Rezlescu N., Doroftei C., Rezlescu E., Popa P.D. (2006). The influence of Sn^4+^ and/or Mo^6+^ ions on the structure, electrical and gas sensing properties of Mg-ferrite. Phys. Stat. Sol. A.

[48] Rusu G.I., Airinei A., Rusu M., Prepeliţă P., Marin L., Cozan V., Rusu I.I. (2007). On the electronic transport mechanism in thin films of some new poly(azomethine sulfone)s. Acta Mater..

[49] Leontie L., Danac R., Carlescu A., Doroftei C., Rusu G.G., Tiron V., Gurlui S., Susu O. (2018). Electric and optical properties of some new functional lower-rimsubstituted calixarene derivatives in thin films. Appl. Phys. A.

[50] Rezlescu E., Doroftei C., Rezlescu N., Popa P.D. (2008). Preparation, structure and gas-sensing properties of gamma-Fe_2_O_3_ and gamma-Fe_2_O_3_-TiO_2_ thick films. Phys. Stat. Sol. A.

[51] Doroftei C. (2016). Formaldehyde sensitive Zn-doped LPFO thin films obtained by rf sputtering. Sens. Actuators B.

[52] Doroftei C., Popa P.D., Rezlescu. N. (2010). The influence of the heat treatment on the humidity sensitivity of magnesium nanoferrite. J. Optoelectron. Adv. Mater..

[53] Doroftei C., Leontie L. (2021). Porous Nanostructured Gadolinium Aluminate for High-Sensitivity Humidity Sensors. Materials.

[54] Rusu G.I., Căplănuş I., Leontie L., Airinei A., Butuc E., Mardare D., Rusu I.I. (2001). Studies on the electronic transport properties of some aromatic polysulfones in thin films. Acta Mater..

[55] Sta I., Jlassi M., Hajji M., Boujmil M.F., Jerbi R., Kandyla M., Kompitsas M., Ezzaouia H. (2014). Structural and optical properties of TiO_2_ thin films prepared by spin coating. J. Sol-Gel Sci. Technol..

[56] Kaiser C., Sandberg O.J., Zarrabi N., Li W., Meredith P., Armin A. (2021). A universal Urbach rule for disordered organic semiconductors. Nat. Commun..

[57] Pancove J. (1979). Optical Processes in Semiconductors.

[58] Erdoğan E., Gündüz B. (2016). Controlling of the optical properties of the solutions of the PTCDI-C8 Organic Semiconductor, Electron. Mater. Lett..

[59] Yakuphanoglu F. (2008). Electrical conductivity, optical and metal–semiconductor contact properties of organic semiconductor based on MEH-PPV/fullerene blend. J. Phys. Chem. Solids.

[60] Yadav B.C., Yadav R.C., Dwivedi P.K. (2010). Sol-gel processed (Mg–Zn–Ti) oxide nano-composite film deposited on prism base as an opto-electronic humidity sensor. Sens. Actuators B Chem..

[61] Moss T.S., Burrell G.J., Elis B. (1973). Semiconductors Opto-Electronics.

[62] Tauc J. (1973). Optical Properties of Solids.

[63] Danac R., Leontie L., Carlescu A., Rusu G.I. (2012). DC Electric Conduction Mechanism of Some Newly Synthesized Indolizine Derivatives in Thin Films. Mat. Chem. Phys..

